# Hazard-based risk grouping effectively stratifying breast cancer patients in post-irradiation long-term heart diseases: a population-based cohort study

**DOI:** 10.3389/fcvm.2023.980101

**Published:** 2023-04-27

**Authors:** Moon-Sing Lee, Wei-Ta Tsai, Hsuan-Ju Yang, Shih-Kai Hung, Wen-Yen Chiou, Dai-Wei Liu, Liang-Cheng Chen, Chia-Hui Chew, Ben-Hui Yu, Feng-Chun Hsu, Tung-Hsin Wu, Hon-Yi Lin

**Affiliations:** ^1^ Department of Radiation Oncology, Dalin Tzu Chi Hospital, Buddhist Tzu Chi Medical Foundation, Chiayi, Taiwan; ^2^ School of Medicine, Tzu Chi University, Hualien, Taiwan; ^3^ Department of Biomedical Imaging and Radiological Sciences, National Yang Ming Chiao Tung University, Taipei, Taiwan; ^4^ Departments of Radiation Oncology, Hualien Tzu Chi Hospital, Buddhist Tzu Chi Medical Foundation, Chiayi, Taiwan; ^5^ Department of Computer Science and Information Engineering, National Cheng Kung University, Chiayi, Taiwan; ^6^ Department of Biomedical Sciences, National Chung Cheng University, Chia-Yi, Taiwan

**Keywords:** synergic effect, heart failure, cardiovascular disease, breast cancer, epirubicin, radiotherapy

## Abstract

**Background:**

Even though advanced radiotherapy techniques provide a better protective effect on surrounding normal tissues, the late sequelae from radiation exposure to the heart are still considerable in breast cancer patients. The present population-based study explored the role of cox-regression-based hazard risk grouping and intended to stratify patients with post-irradiation long-term heart diseases.

**Materials and methods:**

The present study investigated the Taiwan National Health Insurance (TNHI) database. From 2000 to 2017, we identified 158,798 breast cancer patients. Using a propensity score match of 1:1, we included 21,123 patients in each left and right breast irradiation cohort. Heart diseases, including heart failure (HF), ischemic heart disease (IHD), and other heart diseases (OHD), and anticancer agents, including epirubicin, doxorubicin, and trastuzumab, were included for analysis.

**Results:**

Patients received left breast irradiation demonstrated increased risks on IHD (aHR, 1.16; 95% CI, 1.06–1.26; *p* < 0.01) and OHD (aHR, 1.08; 95% CI, 1.01–1.15; *p* < 0.05), but not HF (aHR, 1.11; 95% CI, 0.96–1.28; *p* = 0.14), when compared with patients received right breast irradiation. In patients who received left breast irradiation dose of >6,040 cGy, subsequent epirubicin might have a trend to increase the risk of heart failure (aHR, 1.53; 95% CI, 0.98–2.39; *p* = 0.058), while doxorubicin (aHR, 0.59; 95% CI, 0.26–1.32; *p* = 0.19) and trastuzumab (aHR, 0.93; 95% CI, 0.33–2.62; *p* = 0.89) did not. Older age was the highest independent risk factor for post-irradiation long-term heart diseases.

**Conclusion:**

Generally, systemic anticancer agents are safe in conjunction with radiotherapy for managing post-operative breast cancer patients. Hazard-based risk grouping may help stratify breast cancer patients associated with post-irradiation long-term heart diseases. Notably, radiotherapy should be performed cautiously for elderly left breast cancer patients who received epirubicin. Limited irradiation dose to the heart should be critically considered. Regular monitoring of potential signs of heart failure may be conducted.

## Introduction

Cardiovascular diseases (CVDs), including heart disease, cerebrovascular disease, atherosclerosis, and aortic aneurysm/dissection, are recognized as potential long-term sequelae in cancer survivors (1, 2). Among 28 cancer diseases, 38.0% of patients died from cancer, and 11.3% died from CVDs. Breast cancer patients demonstrated a higher (11.7%) than average (11.3%) risk of death from CVDs (2). Regarding CVDs, 76.3% of deaths were due to heart disease (2).

The risk of treatment-associated heart diseases in breast cancer patients is a particular issue in current clinical medicine. Anthracycline chemotherapy, targeted therapy, and radiation therapy are treatment modalities at risk for heart diseases (3, 4), and they may limit the overall treatment effectiveness (3, 5). Clinically, treatment-associated cardiotoxicity gains significant concern in breast cancer patients (4, 6–8). However, few studies comprehensively demonstrated the synergic effect of anticancer agents and radiotherapy on heart toxicities in breast cancer patients. Even though advanced irradiation techniques have provided better protective effects for the heart, the risk of long-term heart disease in patients who received left breast irradiation is still a considerable concern. Clinically, a potential synergic effect of anticancer agents and radiotherapy on heart toxicities may exist in patients with left breast irradiation. However, the actual hazard sizes of combined treatments are rarely demonstrated, especially in the real-world setting.

In the present study, we extensively explored the events of long-term heart sequelae, including heart failure, ischemic heart disease, and other heart diseases, such as acute pericarditis, cardiomyopathy, and arrhythmia. Anticancer agents, including epirubicin, doxorubicin, and trastuzumab, were selected to examine the synergic effect of radiotherapy on cardiac toxicities in patients who received left breast irradiation. Notably, hazard-based risk grouping was applied to stratify patients.

## Materials and methods

### Ethic consideration and research database

The present population-based cohort study utilized the national database of the Taiwan National Health Insurance (TNHI). The TNHI database contained comprehensive information, including the records of diagnosis and treatment of approximately 99% of people in Taiwan (9), and this database was evaluated strictly and regularly by the National Health Insurance Administration (NHIA) (5). The Institute Review Board (IRB) of the Dalin Tzu-Chi Hospital, Buddhist Tzu Chi Medical Foundation, approved the protocol before the study initiation (B10404014). The IRB waived the requirement for informed consent due to the absolutely de-identified data nature.

**Figure 1:**
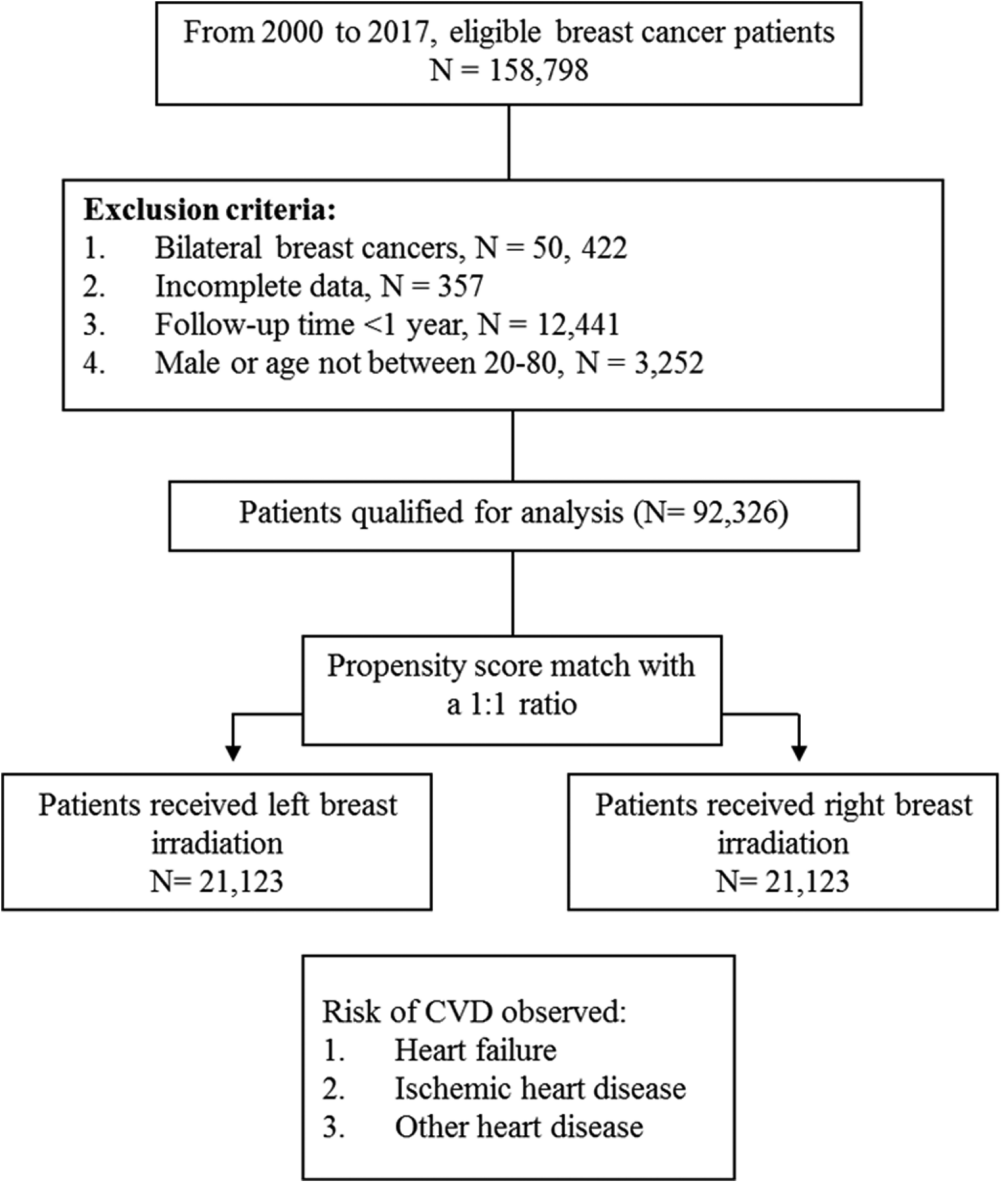
Patient flow chart.

This is a 17-year long-term follow-up cohort study. Figure 1 presents the patient flow chart. From 2000 to 2017, female patients diagnosed with breast cancer aged 20–80 were identified from the TNHI database. We excluded patients with incomplete data, non-irradiation, and a follow-up period of less than 1 year. For balancing the pre-analysis patient population, we used a propensity score match (PSM) to pair patients into two groups: left and right breast irradiation. The match-paired ratio was 1: 1. The PSM is a statistical method based on multiple regression analysis. In this study, we used the basic characteristics of the subjects as independent variables, including age, radiation doses, clinical and pathological stage, surgery type, chemotherapy, hormone therapy, ant-cancer agents, comorbidities, family income, urbanization level, geographic region, and the group as the dependent variable, left-sided and right-sided breast irradiation groups (see Table 1). Each subject was assigned a propensity score, which represents the probability of being assigned to either left-sided or right-sided breast irradiation group, and we further utilized the propensity scores of each subject to perform two-group matching in order to reduce bias caused by potential confounding factors. The detail of propensity score calculated was as follows:

1. Individual propensity score (PS) was calculated from a multivariate Logistic regression model with response variable laterality coded as 0 for the right and 1 for the left while predictors including all the confounding factors listed in the baseline characteristic summary table (see Table 1). The PS indicated how likely an individual with the given covariates was a sample from the cohort of laterality 1.
2. For each individual with a value of PS, for example, ps1 in cohort of laterality 1, an individual from cohort of laterality 0 with PS value closest to the ps1 was selected as a match. Random selection was made when tied. The distance of the two PS values must be ≤0.0001; otherwise, no match was made.

**Table 1:** Patient characteristics.

Variables	Breast cancer patients received radiotherapy after propensity score 1:1 match		Absolute standardized mean difference
	Right	Left	
	*N* = 21,123 (%)	*N* = 21,123 (%)	
Age group (years)			0.0014
20–46	6,890 (32.6)	6,918 (32.8)	
47–53	6,024 (28.5)	6,011 (28.5)	
54–62	5,346 (25.3)	5,336 (25.3)	
>62	2,863 (13.6)	2,858 (13.5)	
RT dose (cGy)			0.0017
3,000–5,040	6,328 (30.0)	6,311 (29.9)	
5,040–6,040	9,755 (46.2)	9,780 (46.3)	
>6,040	5,040 (23.9)	5,032 (23.8)	
C-stage			0.0004
0	1,433 (6.8)	1,440 (6.8)	
I	6,642 (31.4)	6,637 (31.4)	
II	7,871 (37.3)	7,888 (37.3)	
III	2,118 (10.0)	2,114 (10.0)	
IV	640 (3.0)	641 (3.0)	
Unknown	2,419 (11.5)	2,403 (11.4)	
P-stage			0.0018
0	1,640 (7.8)	1,635 (7.7)	
I	7,090 (33.6)	7,077 (33.5)	
II	6,778 (32.1)	6,797 (32.2)	
III	4,490 (21.3)	4,480 (21.2)	
IV	434 (2.1)	434 (2.1)	
Unknown	691 (3.3)	700 (3.3)	
Chemotherapy	14,946 (70.8)	14,965 (70.9)	0.0020
Hormone therapy	15,048 (71.2)	15,098 (71.5)	0.0053
Anti-cancer agents			
Doxorubicin	3,964 (18.8)	3,953 (18.7)	0.0015
Epirubicin	8,622 (40.8)	8,661 (41.0)	0.0037
Trastuzumab	2,154 (10.2)	2,154 (10.3)	0.0043
Docetaxel	6,756 (32.0)	6,803 (32.2)	0.0049
Paclitaxel	1,441 (6.8)	1,432 (6.8)	0.0016
Carboplatin	55 (0.3)	50 (0.2)	0.0040
Cyclophosphamide	14,175 (67.1)	14,192 (67.2)	0.0017
Fluorouracil	9,415 (44.6)	9,432 (44.7)	0.0016
Methotrexate	1,221 (5.8)	1,199 (5.7)	0.0043
Surgery type			0.0014
BCS	12,616 (59.7)	12,603 (59.7)	
MRM	6,401 (30.3)	6,413 (30.4)	
None	2,106 (10.0)	2,107 (10.0)	
Comorbidities			
Hypertension	3,464 (16.4)	3,506 (16.6)	0.0054
Diabetes mellitus	1,605 (7.6)	1,610 (7.6)	0.0008
Family income (NTD per month)			0.0012
<20,100	4,275 (20.2)	4,277 (20.3)	
20,101–22,800	6,556 (31.0)	6,541 (31.0)	
22,801–42,000	5,253 (24.9)	5,268 (24.9)	
>42,000	5,039 (23.9)	5,037 (23.9)	
Urbanization level			0.0011
City	5,629 (26.7)	5,619 (26.6)	
Satellite cities	11,099 (52.5)	11,092 (52.5)	
Rural areas	4,395 (20.8)	4,412 (20.9)	
Geographic region			0.0010
North	10,631 (50.3)	10,641 (50.4)	
Central	4,741 (22.4)	4,747 (22.5)	
South	5,336 (25.3)	5,326 (25.2)	
East	415 (2.0)	409 (1.9)	

RT, radiotherapy; C-stage, clinical stage; P-stage, pathological stage; BCS, breast conserving surgery; MRM, modified radical mastectomy; NTD, New Taiwan dollar; Insurance premium for National Health Insurance is according to family income. Absolute standardized mean difference less than 0.1 is considered as covariates balance between groups after propensity score matching.

After a 1:1 propensity score match for two groups, there were 21,123 patients were included in the left and right breast irradiation group, respectively.

Three systemic anticancer agents, including epirubicin, doxorubicin, and trastuzumab, were selected to investigate each drug's independent effect under different radiation dose levels. Other agents, such as docetaxel, paclitaxel, carboplatin, cyclophosphamide, fluorouracil, and methotrexate, were included as covariates for analysis to examine their potential association with the risk of heart diseases. Radiation doses were defined into three levels, ranging from 3,000 to 5,040 cGy, from 5,040 to 6,040 cGy, and >6,040 cGy. According to the International Classification of Disease, Ninth Revision, Clinical Modification (ICD-9-CM codes), heart diseases are categorized as a diagnosis of heart failure (HF), ischemic heart disease (IHD), and other heart diseases (OHD). The code of ICD-9-CM for heart failure was 428. The code for ischemic heart disease was 410–414, including acute and subacute myocardial infarction. We defined the codes for other heart diseases, including pericarditis (420), endocarditis (421), other diseases of pericarditis and endocarditis (423–424), myocarditis (422), cardiomyopathy (425), conduction disorders (426), cardiac dysrhythmias (427), and ill-defined descriptions and complications of heart disease (429).

Other factors, including age, diabetes mellitus (including type I and type II), hypertension, surgery type, clinical and pathological staging, chemotherapy, and hormone therapy, were also applied as covariates for data analysis. In addition, socioeconomic variables, including geographic region, urbanization level, and monthly income-based insurance premiums, were included in the analysis to reduce bias from lifestyle.

### Statistical analysis

Cox proportional hazard regression was performed to estimate the adjusted hazard ratio (aHR) with a 95% confidence interval (CI) to examine the independent effect of left breast irradiation, when compared with right breast irradiation, on the risk of heart diseases.

In addition, Cox regression was also utilized to estimate the individual hazard based on all covariates for risk grouping and explore the synergic effect of anticancer agents and irradiation dose on heart disease risk. We estimated individual hazard by using the COX regression method with the following formula: y = exp (aX + bY + cZ….); a, b, c = log (aHR); X, Y, Z = covariate variable. All included patients were allocated equally in number into 2, 3, 4, or 5 sub-groups, according to the order of each individual hazard ratios, which were based on parameters including all covariates and socioeconomic variables to assess the risks of IHD, OHD, and HF. The 1/2, 1/3, 1/4, and 1/5 risk subgroups denote the lowest risk subgroup, while the 2/2, 3/3, 4/4, and 5/5 subgroups denote the highest risk subgroup.

A two-sided *p*-value of <0.05 was considered statistically significant. The SAS software (version 9.2; SAS Institute, Inc., Cary, NC) was used for all statistical analyses.

## Results

From 2000 to 2017, 158,798 breast cancer patients were identified, and 92,326 cases were qualified (Figure 1). After a 1:1 propensity score match, 42,246 patients with left (*n* = 21,123) and right (*n* = 21,123) breast irradiation were included for final analysis. The mean ages of the two cohorts who received left and right breast irradiation were 51.20 and 51.17 years old, respectively. After PSM, the two cohorts' pre-analysis clinical and demographic variables are comparable (Table 1).

Table 2 presents the aHRs of CVD after adjusting covariates. Patients with left breast irradiation had trends in risks of ischemic heart disease (aHR, 1.16; 95% CI, 1.06–1.26; *p* < 0.01) and other heart diseases (aHR, 1.08; 95% CI, 1.01–1.15; *p* < 0.05), but not heart failure (aHR, 1.11; 95% CI, 0.96–1.28; *p* = 0.14). Regarding the risk of all heart diseases, the adjusted hazard ratio showed an increasing curve with age. Patients aged >62 years tended to have elevated risks of HF (aHR, 4.11; 95% CI, 3.22–5.24), IHD (aHR, 4.26; 95% CI, 3.65–4.98), and OHD (aHR, 2.34; 95% CI, 2.09–2.62), respectively.

**Table 2:** Adjusted hazard ratios for heart diseases in breast cancer patients received radiotherapy.

	Heart failure (HF)		Ischemic heart disease (IHD)		Other heart diseases (OHD)	
	aHR	95% CI	aHR	95% CI	aHR	95% CI
Left breast RT (right breast RT as ref.)	1.11	0.96–1.28	1.16**	1.06–1.26	1.08*	1.01–1.15
Age (20–47 as ref.)						
47–53	1.58***	1.25–1.99	2.01***	1.75–2.32	1.33***	1.21–1.46
54–62	2.05***	1.63–2.58	2.88***	2.51–3.32	1.63***	1.48–1.80
>62	4.11***	3.22–5.24	4.26***	3.65–4.98	2.34***	2.09–2.62
RT dose (3,000–5,040 as ref.)						
5,040–6,040	0.88	0.73–1.08	0.93	0.82–1.05	0.89*	0.81–0.98
>6,040	0.84	0.67–1.06	1.00	0.87–1.15	1.01	0.90–1.12
C-stage (stage 0 as ref.)						
I	0.94	0.59–1.50	1.00	0.80–1.26	1.16	0.97–1.40
II	0.92	0.58–1.47	0.95	0.76–1.21	1.04	0.86–1.27
III	1.33	0.80–2.18	0.88	0.66–1.16	1.18	0.95–1.46
IV	1.02	0.41–2.54	0.75	0.41–1.38	1.04	0.67–1.60
P-stage (stage 0 as ref.)						
I	1.37	0.86–2.18	1.03	0.83–1.28	1.02	0.86–1.22
II	1.80*	1.11–2.90	0.97	0.77–1.22	0.99	0.82–1.20
III	1.88*	1.14–3.09	0.99	0.77–1.28	1.08	0.88–1.33
IV	1.61	0.59–4.37	0.63	0.30–1.32	0.96	0.59–1.56
Chemotherapy	1.28	0.84–1.95	0.77	0.56–1.05	0.89	0.71–1.11
Anti-hormone therapy	0.95	0.81–1.12	0.87**	0.78–0.95	0.88***	0.81–0.95
Systemic anti-cancer agents						
Doxorubicin	1.09	0.80–1.50	0.99	0.81–1.20	1.02	0.87–1.18
Epirubicin	1.40*	1.04–1.89	0.92	0.76–1.11	1.08	0.93–1.24
Trastuzumab	1.10	0.86–1.42	0.90	0.74–1.08	1.06	0.93–1.22
Docetaxel	0.97	0.79–1.20	0.98	0.85–1.12	0.87**	0.78–0.96
Paclitaxel	0.98	0.74–1.31	1.12	0.92–1.36	0.96	0.82–1.11
Carboplatin	0.82	0.20–3.31	0.53	0.13–2.15	1.19	0.59–2.40
Cyclophosphamide	0.69	0.46–1.02	1.06	0.77–1.46	1.03	0.82–1.30
Fluorouracil	1.04	0.84–1.28	1.12	0.91–1.39	1.01	0.85–1.19
Methotrexate	0.89	0.62–1.29	1.12	0.92–1.36	0.96	0.82–1.11
Surgery type (BCS as ref.)						
MRM	1.12	0.89–1.41	1.07	0.92–1.24	1.13*	1.01–1.26
None	1.14	0.83–1.56	1.07	0.90–1.26	1.16*	1.01–1.33
Comorbidities						
Hypertension	1.75***	1.48–2.07	1.78***	1.61–1.97	1.44***	1.32–1.57
Diabetes mellitus	1.79***	1.48–2.18	1.49***	1.31–1.68	1.16**	1.04–1.30
Family income (NTD per month) (<20,100 as ref.)						
20,101–22,800	0.87	0.72–1.05	0.87*	0.77–0.97	0.88**	0.80–0.97
22,801–42,000	0.81	0.66–1.01	0.80***	0.70–0.90	0.88*	0.79–0.97
>42,000	0.84	0.68–1.04	0.80***	0.70–0.91	0.91	0.82–1.00
Urbanization level (City as ref.)						
Satellite cities	0.98	0.82–1.17	0.90*	0.81–0.99	0.89**	0.82–0.97
Rural areas	1.32*	1.06–1.65	1.04	0.90–1.91	0.98	0.88–1.10
Geographic region (North, as ref.)						
Central	0.85	0.70–1.04	1.05	0.93–1.18	0.94	0.86–1.03
South	0.76	0.62–0.92	0.91	0.81–1.02	0.85***	0.78–0.93
East	1.42	0.95–2.13	0.92	0.67–1.26	1.12	0.89–1.41

RT, radiotherapy; aHR, adjusted hazard ratio; 95% CI, 95% confidence interval; C-stage, clinical stage; P-stage, pathological stage; BCS, breast conserving surgery; MRM, modified radical mastectomy; NTD, New Taiwan Dollar; ref., reference (HR = 1).

* *p *< 0.05.

** *p *< 0.01.

*** *p *< 0.001.

Surprisingly, radiation dosage did not statistically significantly influence the risk of heart diseases (Table 2). On the other hand, among anticancer drugs, epirubicin was statistically significantly associated with an increased risk of heart failure (aHR, 1.40; 95% CI, 1.04–1.89; *p* < 0.05), but not doxorubicin and trastuzumab. No significant relationship was presented regarding other systemic anticancer agents such as docetaxel, paclitaxel, carboplatin, cyclophosphamide, fluorouracil and methotrexate for the risks of heart failure, ischemic heart disease and other heart diseases.

Regarding the influence of comorbidities, both hypertension and diabetes mellitus were markedly associated with high risks of heart diseases (for heart failure, aHR of 1.75 and 1.79; for ischemic heart disease, 1.78 and 1.49; for other heart diseases, 1.44 and 1.16, respectively).

For socioeconomic variables, including family income, urbanization level, and geographic region, only rural areas were statistically significantly associated with an increased risk of heart diseases. Breast cancer patients who lived in rural areas had a higher risk of heart failure (aHR, 1.32; 95% CI, 1.06–1.65; *p* < 0.05) than those who lived in the city. However, there was no apparent influence from different urbanization levels on ischemic and other heart diseases.

Incidentally, we observed that anti-hormone therapy seemly showed risk reduction in IHD (aHR, 0.87; 95% CI, 0.78–0.95) and OHD (aHR, 0.88; 95% CI, 0.81–0.95; Table 2).

**Figure 2:**
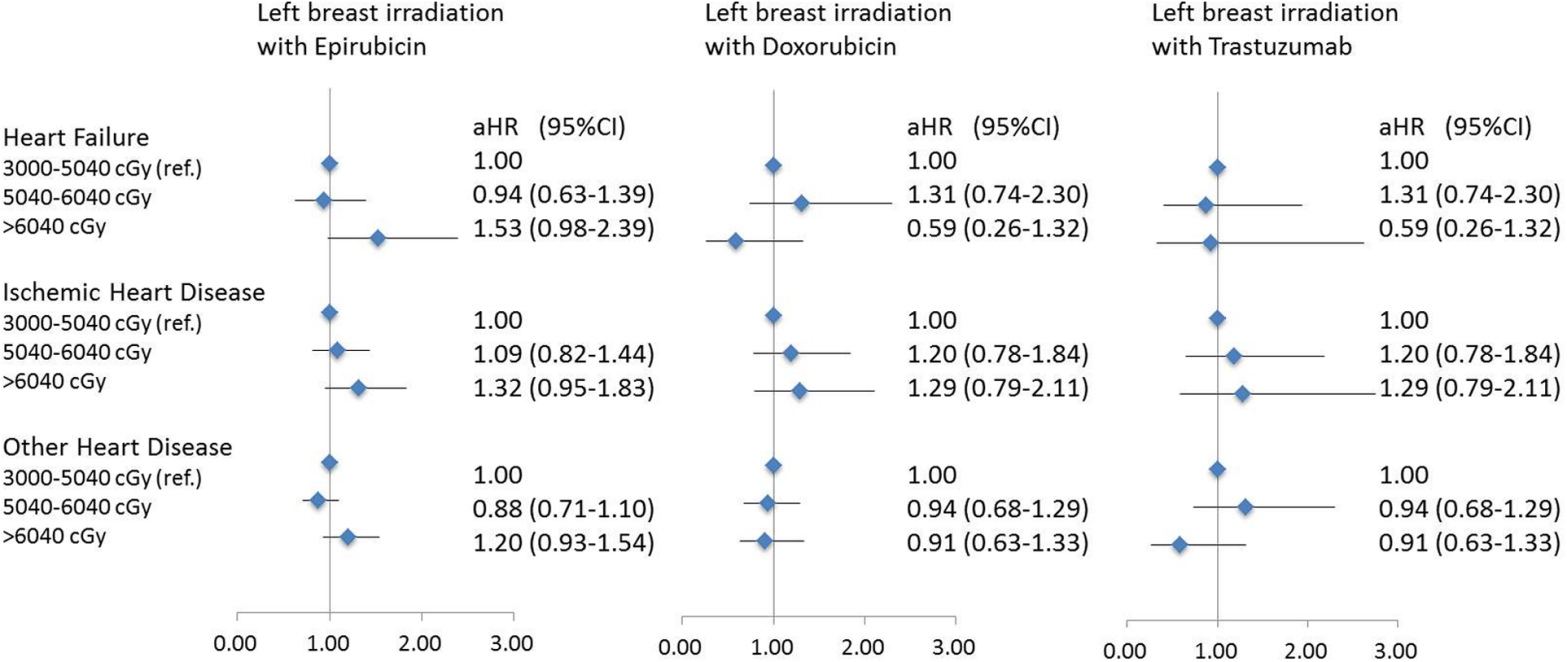
A forest plot for demonstrating heart disease risk in patients with left breast irradiation according to three main systemic anti-cancer agents and different radiation dose.

Figure 2 demonstrated the risk of CVDs in breast cancer patients who received left breast irradiation by each anticancer agent, i.e., epirubicin, doxorubicin, and trastuzumab. Notably, patients who received left breast irradiation dosage of >6,040 cGy, epirubicin increased heart failure risk, which reached a marginal statistical significance (aHR, 1.53; 95% CI, 0.98–2.39; *p* = 0.058), while doxorubicin (aHR, 0.59; 95% CI, 0.26–1.32; *p* = 0.19) and trastuzumab (aHR, 0.93; 95% CI, 0.33–2.62; *p* = 0.89) did not. Besides, there was no similar finding for the risk of ischemic and other heart diseases. This observation indicated a particular association between epirubicin and left breast irradiation on the risk of heart failure.

Table 3 shows hazard-based risk sub-grouping. According to the order of the individual hazard, all included patients were allocated equally in number into 2, 3, 4, or 5 sub-groups based on parameters including all covariates and socioeconomic variables showed in Table 2 to assess the risks of IHD, OHD, and HF.

**Table 3:** Hazard-based risk-group stratification.

Divided to subgroups^a^		IHD (*N*)	OHD (*N*)	HF (*N*)
Two risk groups	1/2	21,132	21,122	21,120
	2/2	21,114	21,124	21,126
Three risk groups	1/3	14,081	14,083	14,082
	2/3	14,054	14,078	14,081
	3/3	14,111	14,085	14,083
Four risk groups	1/4	10,566	10,562	10,561
	2/4	10,566	10,560	10,559
	3/4	10,553	10,562	10,564
	4/4	10,561	10,562	10,562
Five risk groups	1/5	8,450	8,450	8,449
	2/5	8,449	8,448	8,449
	3/5	8,449	8,449	8,450
	4/5	8,448	8,450	8,449
	5/5	8,450	8,449	8,449

IHD, ischemic heart disease; OHD, other heart disease; HF, heart failure; N, patient number. Note that the each patient's individual hazard is estimated according to Table 2. The individual hazard is estimated by using the COX regression method with the following formula: y = exp (aX + bY + cZ….); a, b, c = log (aHR); X, Y, Z = covariate variable. According to the order of the individual hazard, all included patients were allocated equally in number into 2, 3, 4, or 5 sub-groups.

^a^ The 1/2, 1/3, 1/4, and 1/5 risk subgroups denote the lowest risk subgroup, while the 2/2, 3/3, 4/4, and 5/5 subgroups denote the highest risk subgroup.

**Figure 3:**
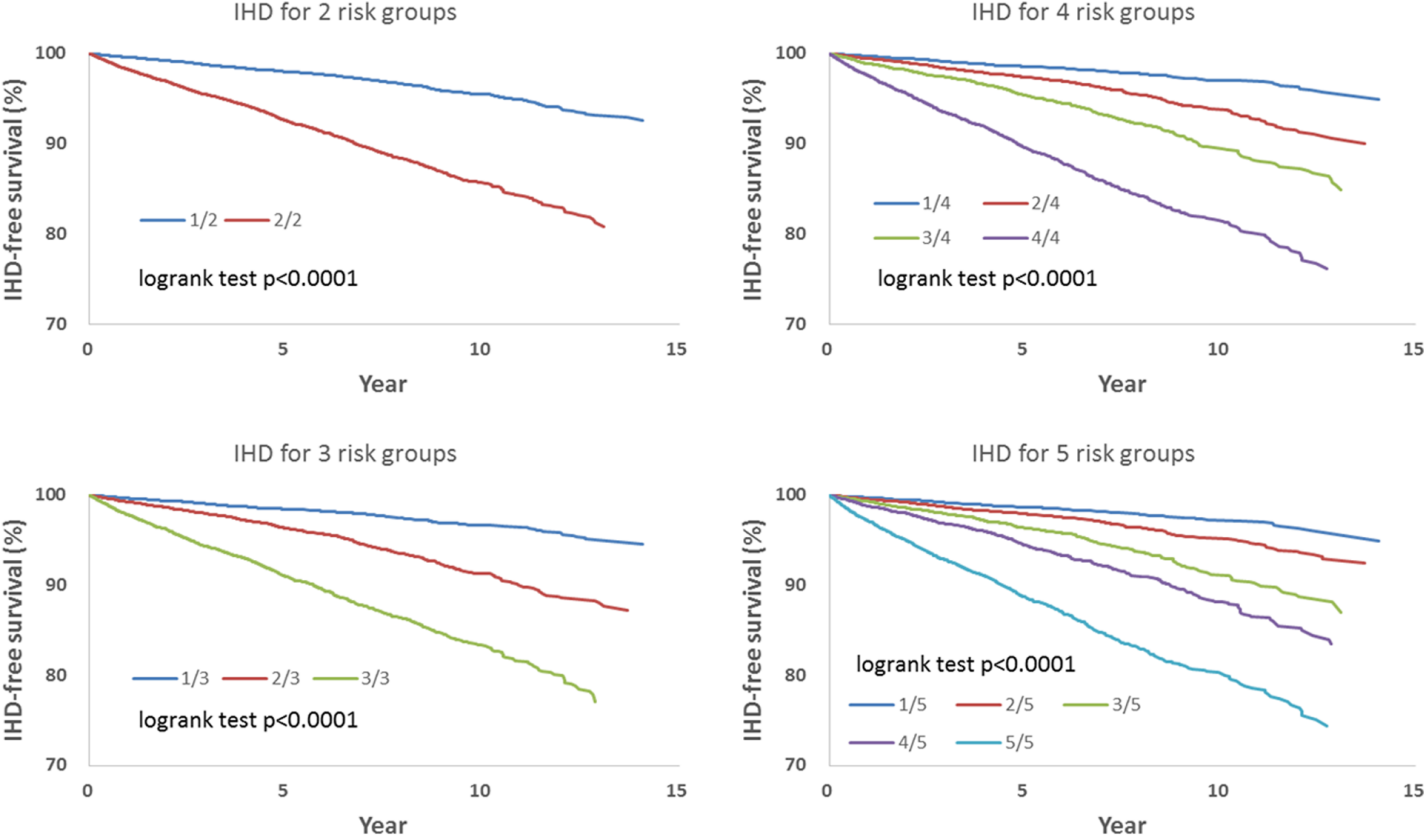
Based on Tables 2, 3, the hazard-based risk grouping effectively stratified irradiated breast cancer patients in the endpoint of ischemic heart disease (IHD, *p* < 0.0001 in all sub-grouping). Note that the each patient's individual hazard is estimated according to Table 2. According to the order of the individual hazard, all included patients were allocated equally in number into 2, 3, 4, or 5 sub-groups based on parameters including all covariates and socioeconomic variables showed in Table 2 to assess the risks of IHD, OHD, and HF.

**Figure 4:**
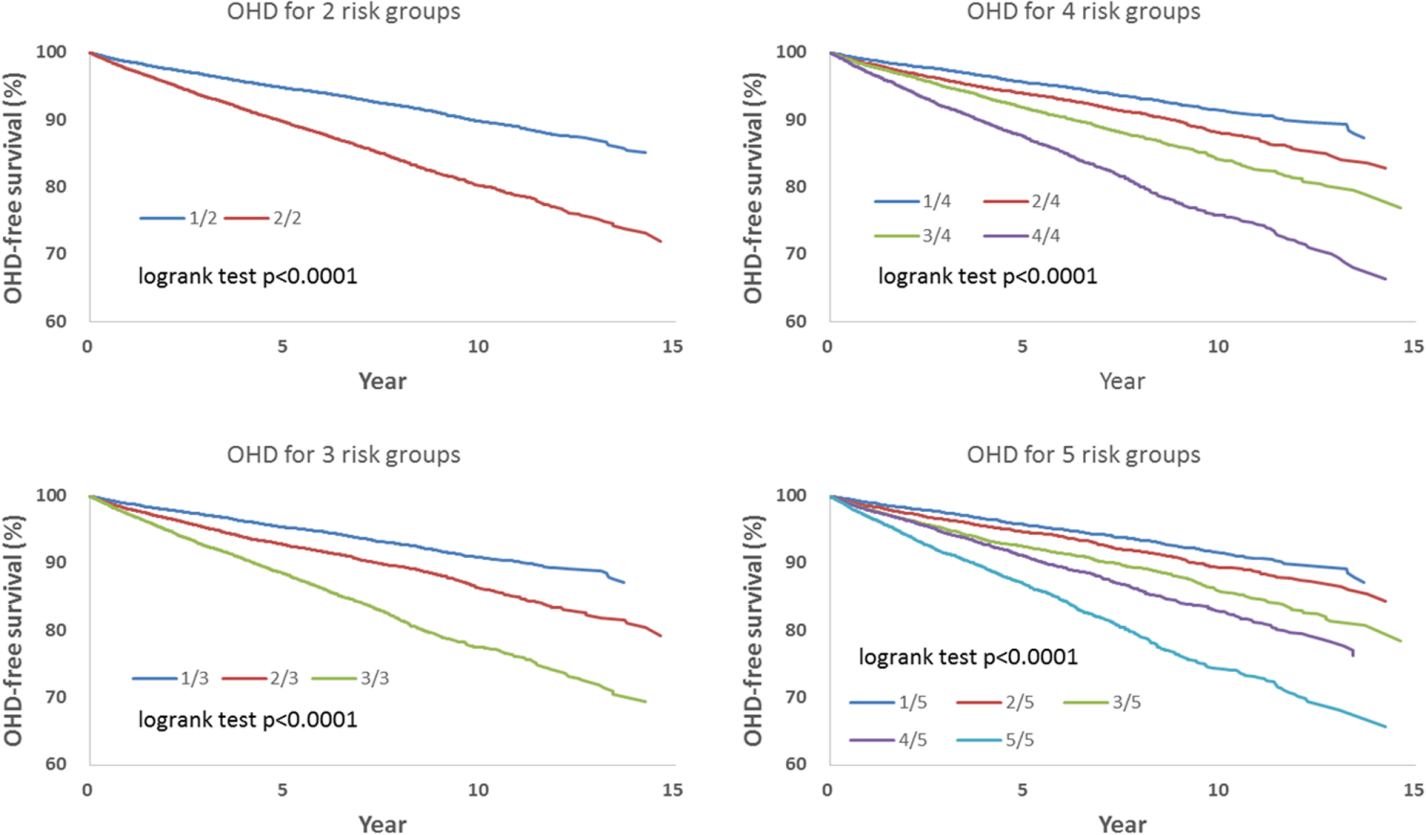
Based on Tables 2, 3, the hazard-based risk grouping effectively stratified irradiated breast cancer patients in the endpoint of other heart diseases (OHD; *p* < 0.0001 in all sub-grouping).

**Figure 5:**
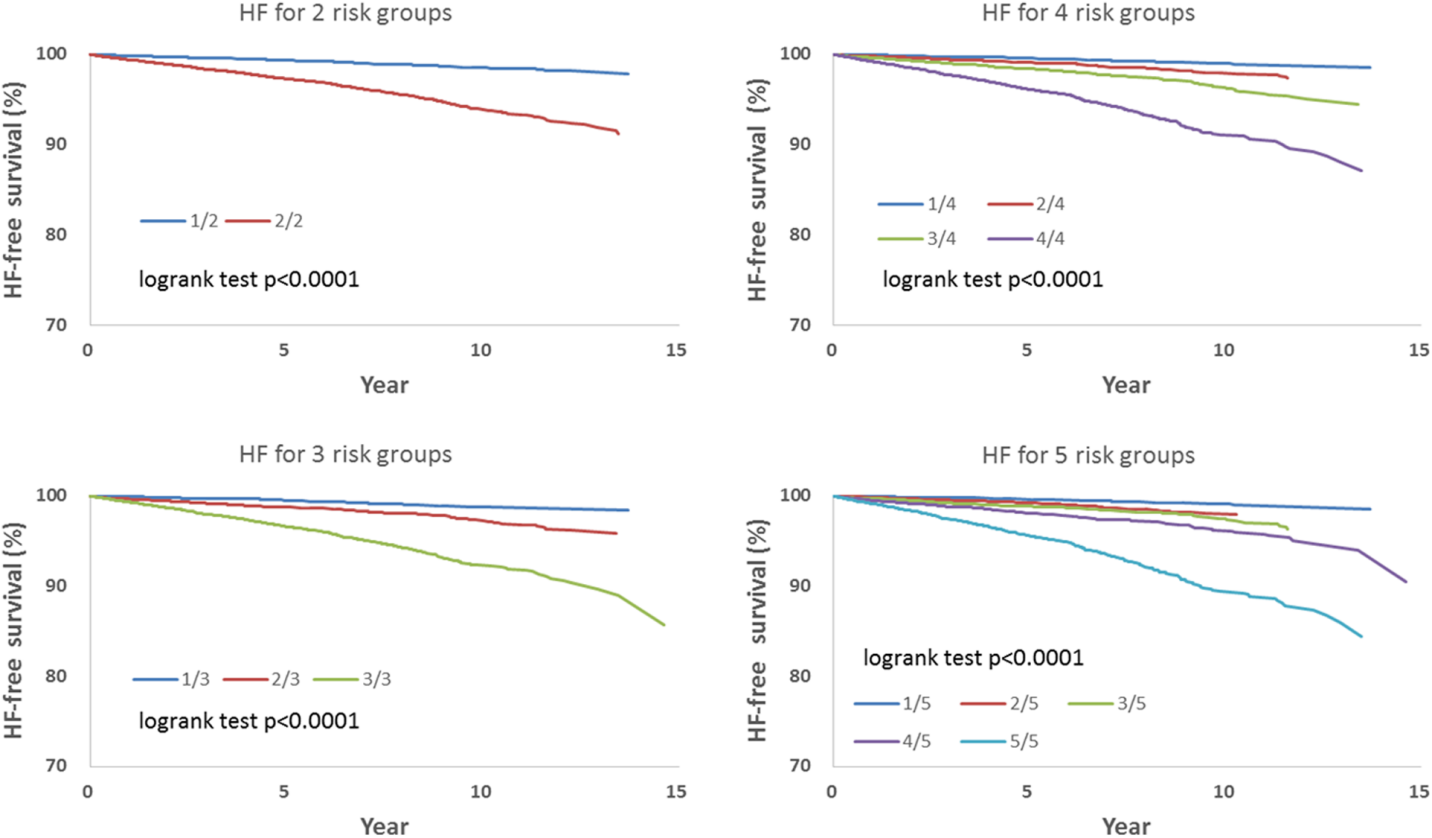
Based on Tables 2, 3, the hazard-based risk grouping effectively stratified irradiated breast cancer patients in the endpoint of heart failure (HF; *p* < 0.0001 in all sub-grouping).

We observed that hazard-based risk grouping effectively stratified irradiated breast cancer patients in the endpoints of IHD (Figure 3), OHD (Figure 4), and HF (Figure 5). Note that statistical significances are found in two, three, four, and five risk-grouping in all three types of heart diseases (*p* < 0.0001).

## Discussion

The present population-based cohort study utilized Cox regression to estimate hazards for risk grouping and stratify breast cancer patients in post-irradiation long-term heart diseases. The risk of treatment-associated heart disease from the synergic effect of anticancer drugs and left-sided breast irradiation was also examined. Our study confirmed the previous observation that breast cancer patients had an elevated risk of treatment-associated heart disease (3, 10), especially for those who received left-sided irradiation (3, 11, 12). Moreover, when it comes to the synergic effect of treatment-associated cardiac injury, the role of epirubicin and left-sided breast irradiation in heart failure is highlighted in the present study, especially in the elderly population. Among breast cancer patients who were given both epirubicin and radiation therapy, the risk of heart failure was elevated when left-sided irradiation with a total dose of >6,040 cGy was given. Our observation suggested that among drugs of anticancer therapy, epirubicin may increase the effect of radiotherapy-associated cardiac injury in breast cancer survivors.

A well-known population-based case-control study of breast cancer patients in Sweden and Denmark by Darby et al. demonstrated that major coronary events increased linearly with the dosage of irradiation to the heart, increasing by 7.4% (95% CI = 2.9–14.5, *p* < 0.001) per gray in the mean radiation dose, and they found the significant radiation-related increase in the risk of ischemic heart disease (10). Similar to the study of Darby et al., our results found a high risk of IHD and OHD when left-sided breast irradiation was given (Table 2). Indeed, the increased risks of coronary heart disease and myocardial infarction observed evidently in patients received left-sided irradiation have been reported previously (13–15). Radiation therapy for left-sided breast cancer patients may cause irradiation-associated perfusion defects and possible wall-motion abnormalities (16). The anterior portions of the left ventricle were indicated to associate with wall-motion defects, which correspond to the heart region within the radiotherapy field (16). In addition, it has been reported that receiving left-sided breast irradiation may result in high-grade stenosis of the left-anterior artery (LAD) when compared with right-sided breast irradiation (17), which cannot be avoided entirely during radiotherapy.

Compared with ischemic heart disease or other heart diseases, the risk of heart failure may be more susceptible to the anthracycline chemotherapy agents (18, 19). Compared with other anticancer drugs, our study showed that epirubicin was statistically significantly related to the risk of heart failure (Table 2) instead of the risk of ischemic heart disease and other heart diseases. More interestingly, the synergic effect of epirubicin-induced and left-sided breast irradiation-induced on heart failure was found in our study. The risk of heart failure emerged as the role of epirubicin under a high dosage of radiotherapy was considered (Figure 2). This observation suggested that the risk of treatment-associated heart failure may be multifactorial; the risk of anthracycline-induced cardiac dysfunction needs to be noted when patients who were under the circumstance of a high dosage of left-sided breast irradiation. Although epirubicin has been announced with a low cardiac toxicity profile (20, 21), epirubicin-associated heart failure is still a concern in breast cancer survivors (22, 23). A 20-year follow-up study indicated that the cumulative incidence of heart failure was higher (up to a three-fold increased risk) in the epirubicin treatment group when compared with the non-epirubicin group (22). Although both epirubicin and doxorubicin are members of the anthracycline family and demonstrate the dose-dependent effect of early- and late-onset chronic cardiotoxicity (24–26), clinical trials with the direct head-to-head comparisons between doxorubicin and epirubicin are still needed (clinical trial: NCIC CTG MA.21) (27). Our results demonstrated that the magnitude of radiotherapy-associated cardiac dysfunction may be enhanced by epirubicin when the total dose of radiation was up to >6,040 cGy and increase the risk of heart failure further. The mechanism of this increased long-term risk of heart failure is uncertain, but the insights of mitochondrial dysfunctions (28–30) and the production of free radicals (28, 31) have been proposed.

Trastuzumab did not show a significant association with the risk of CVDs, nor did it have a synergic effect with radiotherapy in patients with left-sided irradiation in the present study. This finding implies that trastuzumab-related cardiovascular events are relatively low and not apparent relative to anthracycline chemotherapy. The HER2 receptors are reported to be expressed in cardiomyocytes (32), and the alteration in cellular metabolic pathways in cardiomyocytes was indicated as a critical mechanism underlying the development of cardiac dysfunction (33). However, it was reported that the cardiac dysfunction associated with trastuzumab mainly occurs during trastuzumab-treatment (34) and is considered reversible (35, 36) and tolerated (37). Besides, having prior anthracycline treatment is identified for trastuzumab-associated cardiotoxicity (6, 38), in which the incidence of cardiac dysfunction was reported to be 4% with trastuzumab alone and 27% with the combination of anthracycline and cyclophosphamide (39). Even with a median follow-up of 3.6 years, severe chronic heart failure in the trastuzumab group remained low at 0.8% (34).

For risk factors associated with the risk of heart diseases, our study showed that the elevated risk of heart diseases was significant across age groups, and high risk existed in patients aged >62 years in particular, with up to the 4-fold increased risk of heart failure and ischemic heart disease (Table 2). Consistent with previous studies (40, 41), the increased age is related to the elevated risk of fatal myocardial infarction after left-sided post lumpectomy radiotherapy. The increased likelihood of mortality had been observed in patients aged 60 years and older (*p* = 0.01) (40). In addition, a previous study showed that preexisting hypertension is highly associated with increased CVDs risk, with the risk ratio for the development of coronary artery disease being 1.59 for left- versus right-irradiated patients (42), which is not far from our reported risk ratio (range, 1.44–1.78). A relationship between a history of diabetes and the risk of CVD-related mortality was reported in breast cancer patients (43). Diabetic patients had significantly high baseline CVD risks (range, 11.8%–24.2%), and the mean 10-year cumulative risk was 3.7% and 3.9% in patients using the DIBH-technique and free-breathing technique, respectively (44). It suggested that caring for breast cancer patients with diabetes should include attention to CVD risk factors (45).

### Strengths and limitations

We comprehensively investigated the risk of heart diseases, including ischemic heart disease, heart failure, and other heart diseases, among patients who received left-sided breast irradiation. The strength of the present study is that this is a nationwide cohort study with a 17-year long-term follow-up in Taiwan. In addition, by examining the risk of heart diseases by each anticancer drug under different levels of left-sided breast irradiation dose, our observation provides further information concerning the synergic effect of chemotherapy- and radiotherapy-associated cardiotoxicity among breast cancer survivors. Finally, we used a propensity score to match all the covariates, including clinical information, comorbidities, and socioeconomic variables, to reduce potential bias before statistical analysis.

Regarding this study's limitation, some information is unavailable in our database. For example, the mean heart dose and other heart dose parameters, such as the mean dose to the left anterior descending artery, are unavailable from our database, which may influence assessing the extent of radiotherapy-associated cardiac injury. In addition, we lack some information in our data analysis, such as a history of tobacco use, body mass index, familial history of myocardial infarction, or other cardiovascular diseases, which are reported as preexisting risk factors related to the incidence of CVDs (1, 14, 46–48).

## Conclusion

Hazard-based risk grouping may help stratify breast cancer patients at risk of post-irradiation long-term heart diseases. Generally, systemic anticancer agents, including chemotherapy and targeted therapy, are safe in conjunction with radiotherapy for managing post-operative breast cancer patients. However, radiotherapy should be performed cautiously for elderly left breast cancer patients who received epirubicin. Decreasing the irradiation dose to the heart should be critically considered, and regular monitoring of potential signs of heart failure may be conducted.

## Data Availability

The raw data supporting the conclusions of this article will be made available by the authors, without undue reservation.
